# Anionic character of the conduction band of sodium chloride

**DOI:** 10.1038/s41467-022-28392-8

**Published:** 2022-02-21

**Authors:** Christopher C. Leon, Abhishek Grewal, Klaus Kuhnke, Klaus Kern, Olle Gunnarsson

**Affiliations:** 1 Max-Planck-Institut für Festkörperforschung, Heisenbergstraße 1, 70569 Stuttgart, Germany; 2 Institut de Physique, École Polytechnique Fédérale de Lausanne, 1015 Lausanne, Switzerland

**Keywords:** Electronic properties and materials, Electronic structure, Surfaces, interfaces and thin films

## Abstract

The alkali halides are ionic compounds. Each alkali atom donates an electron to a halogen atom, leading to ions with full shells. The valence band is mainly located on halogen atoms, while, in a traditional picture, the conduction band is mainly located on alkali atoms. Scanning tunnelling microscopy of NaCl at 4 K actually shows that the conduction band is located on Cl^−^ because the strong Madelung potential reverses the order of the Na^+^ 3*s* and Cl^−^ 4*s* levels. We verify this reversal is true for both atomically thin and bulk NaCl, and discuss implications for II-VI and I-VII compounds.

## Abstract (web summary)

The current work finds that ordinary table salt behaves unexpectedly. The chloride ions alone dominate the electronic states at both edges of its band gap although sodium ions are also present. This is important when NaCl is used as an insulator.

## Introduction

Insulating compounds host a rich and very varied physics. One distinguishes between ionic^1^, Slater^2^, Mott^3^ and charge transfer^4–7^ insulators, all involving quite different mechanisms and having different properties. Slater and Mott insulators primarily involve a partly filled band, in which antiferromagnetic or Coulomb interactions open up a gap. The late transition metal oxides are examples of charge transfer insulators, involving the O 2*p* band and the, partly filled, transition metal 3*d* band. Coulomb interactions are essential also in this case. Ionic insulators, e.g., alkali halides, appear to be conceptually simpler. In a one-particle picture, all bands are completely full or empty, and Coulomb interactions are less essential. There is a large charge transfer from the alkali atoms to the halogen atoms, which become positively and negatively charged ions. The valence band is primarily of halogen *p* character. This band is separated from the conduction band by a large gap. The conduction band is assumed to have mainly alkali character, involving an empty alkali *s* level outside a full shell. While this picture has been presented in many text books and publications^1,8–13^ this view has occasionally been questioned on theoretical grounds. Slater and Shockley^14^ suggested a different picture for NaCl, in which the conduction band also has a substantial Cl 4*s* character. Clark^15,16^, de Boer and de Groot^17,18^, as well as Olsson et al.^19^. performed band structure calculations for NaCl and concluded that the conduction band is actually mainly located on the Cl^−^ ions. These theoretical results, however, seem to have been largely overlooked or ignored, possibly because of problems with uniquely assigning charges to ions.

We present a heuristic calculation which suggests that a very large Madelung potential^20^ (≈9 eV) can reverse the order of the Cl^−^ 4*s* (above the vacuum level) and Na^+^ 3*s* (at ≈ −5 eV) levels and imply a conduction band of mainly Cl character. (The Madelung potential is the potential at any ion position in an ionic crystal due to the combined electrostatic potentials of the infinite number of ions in the crystal.) However, such a calculation (and similar considerations) alone is not decisive proof of this reality because of confounding factors such as the large spatial extent of *s* orbitals and nonunique assignments of charges to ions. For exactly this reason, we perform an experimental study of the conduction band of NaCl using scanning tunnelling microscopy (STM) providing a real space picture of states, which are centred on the Cl^−^ ions across the entire band gap.

## Results

Despite the many STM studies addressing the structure and growth of NaCl films^21–25^, such a detailed study of the NaCl conduction band has not yet been performed, possibly because it is very challenging to obtain atomic resolution on NaCl at positive bias (e.g., *U* > 1 V)^22^. These difficulties preclude a straightforward spatial mapping of the NaCl conduction band by STM and an identification of the relative local density of states at Na and Cl positions. The instability of the tunnelling condition necessary to achieve atomic resolution simply prohibits addressing the conduction band directly.

We circumvent this problem by making the bias very positive to approach the conduction band very closely without exceeding the band gap, and harnessing our understanding of the tunnelling process under these conditions. We present STM images of NaCl(100) on Au(111), varying the bias over a large range. They show that tunnelling happens through the Cl^−^ ions, even for energies just below the conduction band, indicating that the conduction band also has mainly Cl character.

To support this conclusion we present tight-binding (TB) calculations for two models of a NaCl film on a Au surface, with the conduction band mainly on either the Na^+^ or Cl^−^ ions. These calculations show that features in STM images for energies just below the conduction band indeed are located on the same ions as the conduction band itself.

We compare NaCl on Au and bulk NaCl theoretically. We find that the order of the Na 3*s* and the Cl 4*s* levels are reversed by similar amounts in both cases, as discussed extensively in Supplementary Note 2. The conclusions obtained for NaCl on Au should therefore also apply to bulk NaCl.

We furthermore extend the discussion of NaCl to II–VI and other I–VII compounds, suggesting that the conduction band could have a substantial weight on the anion also in these cases.

### STM topography of NaCl over the entire band gap

Because few experimental techniques can characterise the conduction band character of the alkali halides with atomic resolution, we investigated the NaCl(100) surface with STM. Since directly accessing the conduction band of NaCl(100) involves high electric fields that destroy its structural integrity^22^, we do measurements at voltages as close as possible to the conduction band edge while staying within the band gap of NaCl. Topographs are measured at constant current whose colour scale represent tip-sample distance. By repeating the measurements at certain voltages, the tip quality was monitored and care was taken that the relatively high bias voltages did not modify the tip character or shift the tip apex. Similarly, the tip drift over time, typical for STM, was monitored and corrected in the presented data.

**Fig. 1:**
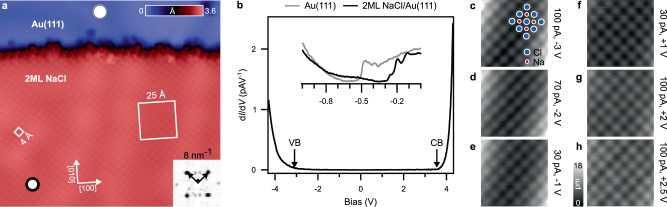
STM of 2 ML NaCl(100) on Au(111) with topography and d*I/*d*V* measurements. **a** Overview of the measurement area (150 × 140Å^2^, *I*_*T*_ = 8 pA, *V*_*S*_ = −50 mV). 25 × 25 Å^2^ region marked, investigated for bias values between −3.0 V and +2.5 V. Inset: Fourier transform of **f** centred over a 8 nm^−1^ × 8 nm^−1^ region in reciprocal space. **b** Differential conductance (d*I/*d*V*) measured atop 2 ML NaCl(100)/Au(111) (marked by the black ring in **a**). The arrows point to onset of valence band (VB) and conduction band (CB). Inset. Surface state features of Au(111) and related interface state of 2 ML NaCl(100)/Au(111) measured at the positions marked in **a** by a grey ring and a black ring, respectively. Vertical scale: arbitrary units. **c**–**h** Raw data showing grayscale topography images obtained for sample bias between −3.0 V and +2.5 V as indicated. The red and blue dots in **c** mark the positions of Na and Cl ions of the NaCl(100) lattice, respectively. The corresponding tunnelling parameters (current, voltage) are indicated next to each topography image.

Figure 1a shows a region of Au(111) covered with a 2 monolayer (ML) thick (apparent height: 309 ± 3 pm) NaCl terrace, consistent with prior studies^26^. Atop the NaCl terrace, the protrusions form a square lattice whose unit cell length is 4 Å (Fig. 1a). The square lattice is reproduced in reciprocal space (Fourier transform inset, Fig. 1a).

We survey the electronic structure of the system at selected positions marked by grey and black rings in Fig. 1a. The pronounced surface state of Au(111) shows an onset at −500 mV measured at the grey ring. This onset shifts to ≈−250 mV when measured on Au-NaCl at the black ring. This shift originates from the Pauli interaction experienced between NaCl and Au(111). NaCl acts as a dielectric, polarises and weakens the image potential arising from Au(111) alone^27,28^ (inset Fig. 1b) measured at the black ring. A large range voltage scan of differential conductance indicating density of states of NaCl is shown in Fig. 1b. Arrows point to the valence and conduction band onsets at −3.0 V and +3.6 V.

The region marked by the white box, in the fcc region of herringbone reconstruction of Au(111), in Fig. 1a is scanned with high stability and signal to noise ratio (Fig. 1c–h) for applied sample voltages from −3 V to +2.5 V, respectively. At applied bias of −3.0 V (Fig. 1c), a voltage close to the valence band edge of NaCl (Fig. 1b), a square lattice of protrusions is seen whose intensity is proportional to the *z*-corrugation. Each protrusion is assigned to Cl. The assignment of protrusions at negative voltage to Cl is in agreement with many earlier studies^19,22,29^. We do not observe any change in position of *z*-corrugation at negative voltages (Fig. 1c–e). These measurements further corroborate the valence band being mainly Cl in character.

We now consider the same region scanned at positive bias. Simple but ultimately misleading electrostatic arguments suggest that the topographic protrusions would become centred over Na^+^ ions. For instance, electron extraction at negative bias is helped at the Cl^−^ positions with the Cl^−^ being relatively electron-rich. Electron injection at positive bias is helped at the Na^+^ positions with the Na^+^ being relatively electron-poor. Thus, one might anticipate that inverting the applied bias polarity should result in an inversion of the topographic contrast due to *z*-corrugation resulting from Na^+^ ions instead of Cl^−^ ions.

We perform this experiment and obtain evidence to the contrary. Fig. 1f–h show the result of probing the NaCl layer at positive bias up to +2.5 V. By comparing these topographic measurements with those obtained at negative bias (Fig. 1c–e), it is evident that the protrusions remain centred over Cl and do not shift over to Na. If the conduction band were cationic in character, one would have expected to see topographic intensity over the Na^+^ ions. Yet no contrast inversion is observed when the bias polarity is inverted. In the band gap of NaCl, the *z*-corrugation is correlated to the electronic states of energetically nearest neighbouring bands. Thus, the experiment shows that the electronic states of NaCl have substantial weight on Cl rather than Na, irrespective of bias polarity. When one tunnels at energies within the band gap of NaCl, electron transport in NaCl is dominated by the role of Cl^−^ ions at both negative and positive bias. This implies that both the valence and conduction bands of NaCl are anionic in character, a point that is to be emphasised.

The expected role of Na^+^ ions in the conduction band of NaCl asserted by many textbooks and publications is simply absent. Rather, we see evidence for Cl functioning as both an electron donor and acceptor in NaCl. We conceptualise the electronic structure of NaCl as that of spectator Na^+^ cations holding together Cl^−^ anions that are mainly responsible for electron conduction.

**Fig. 2:**
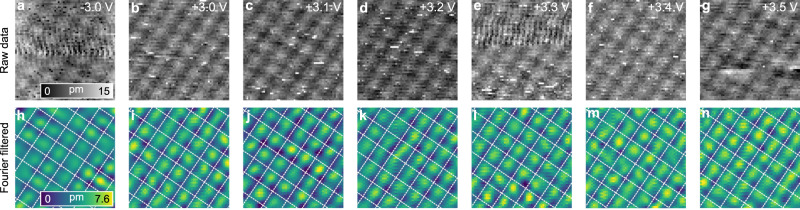
STM of 3 ML NaCl(100) on Au(111) at voltages close to both edges of the band gap. **a**–**g** Raw (grayscale) and **h**–**n** Fourier filtered (coloured) at the indicated voltages, *I*_*T*_ = 15 pA. Scale: 24 × 24 Å^2^. The white dashed lines mark the position of Na ion rows of the NaCl(100) lattice.

To further evidence the markedly anionic (rather than cationic) character of the NaCl conduction band, we expand the voltage range of the measurements to +3.5 V in exchange for greatly reduced tip stability and signal to noise ratio. However, we still keep the absolute piezo drift (0.014 nm min^−1^) during data acquisition to be significantly smaller than the Cl^−^-Na^+^ ion spacing (≈0.3 nm) and along a direction that avoids introducing spurious contrast inversions due to drift. These efforts are shown in Fig. 2 in which a different sample area of 3 ML NaCl(100) (483.5 ± 5.4 pm thickness) on Au(111) is scanned with a newly prepared tip at select voltages and analysed with Fourier filtered scans. As discussed by Lauwaet et al.^26^, the interface state (see inset Fig. 1b) wave function for NaCl(100) on Au(111) does not extend further than two NaCl layers. In the discussion in Supplementary Note 2, we conclude that electronically, 2 ML and 3 ML NaCl are identical. In contrast to Fig. 1, the scans in Fig. 2 are taken across the well-defined fcc and hcp regions of the Au(111) herringbone reconstruction.

At bias >+2 V, the risk of tip changes and spontaneous defect creation in the NaCl layer increase considerably. As seen in Fig. 2a–g, while tip transients do prevent smooth scanning at high bias, they do not completely destroy the important atomic resolution. The low and high frequencies are Fourier filtered to mitigate noise and to clearly highlight the periodic *z*-corrugation. The measurements in Fig. 2 attain atomic resolution on NaCl even at higher bias than the ones in Fig. 1. Once again, at −3 V, *z*-corrugation is assigned to Cl positions and does not shift to Na positions (lattice positions marked by white dashed linse in Fig. 2h–n) at any of the indicated applied voltages in Fig. 2. Repeating these measurements at other voltages between −3.0 V to +3.5 V reveals no evidence for electron density maxima at Na. The results in Fig. 2 show that electron density remains centred over Cl, even up to +3.5 V (Fig. 2g, n) which is quite close to the empirically measured conduction band edge (≈+3.6 V) shown in Fig. 1b. The second data set thus demonstrates that the conclusions about tunnelling being weighted on Cl in NaCl holds more generally and is observed on different NaCl layer heights, on fcc as well as hcp areas and by using a tip after different preparation.

### Tight binding calculations

We now perform tight-binding calculations for a model of a 3 ML NaCl(100) film on a Au(111) surface (see Supplementary Note 1). We use two different sets of parameters for the NaCl film, resulting in a conduction band mainly of either Na 3*s* or Cl 4*s* character. In the first set we use 3*s* and 3*p* basis states on the Cl atoms, and in the second set the Cl 3*s* basis states are replaced by 4*s* basis states. States in the energy gap of NaCl are described as linear combinations of valence and conduction states.

**Fig. 3:**
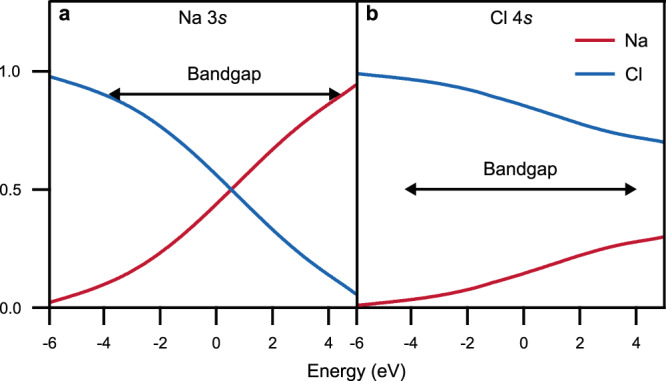
Character of the gap states of NaCl including Madelung potential. Normalised relative weight of gap states on Na and Cl atoms in the outermost NaCl layer as a function of energy, *E*, in or close to the gap (indicated by the arrows). The parameters were adjusted so that the conduction states have mainly **a** Na character and **b** Cl character. The Figure illustrates that electrons travelling through the gap at energies close to the conduction band, have similar character as those in the conduction band.

In Fig. 3a, we use conventional parameters, including 3*s* and 3*p* states on the Cl atoms^12^ and neglect the Madelung potential. (Note, that in the calculation with Cl 3*s* and 3*p* orbitals only, the conduction band is repelled upwards by these states. Including higher states on Cl would tend to have the opposite effect, requiring a higher Na 3*s* level to obtain the correct band gap. This may then require a contribution from the Madelung potential also in this case). As we increase the energy through the gap, the character of the state changes from mainly Cl character close to the valence band to mainly Na character close to the conduction band, in contradiction to the experimental finding.

Figure 3b shows results for the second set of parameters, including 3*p* and 4*s* states on the Cl atoms. In this case, it is essential to include a Madelung potential to obtain a conduction band of mainly Cl character and a correct band gap. The result is that the gap states have mainly Cl character throughout the whole gap, in agreement with experiment. This then provides further evidence that not only the gap states but also the conduction band has mainly Cl character. We emphasise again, the inclusion of Cl 4*s* states in these calculations.

Tunnelling through an insulator band gap proceeds via the electronic states of the energetically nearest neighbouring bands. If NaCl were composed of a Cl^−^ based valence band and a Na^+^ based conduction band, an atomically resolved STM map of it would show voltage-dependent contrast that inverts at a specific voltage in the band gap suggested in Fig. 3a. However, this inversion does not happen in the experiment. In fact, the same type of ion appears bright at energies near both the valence and conduction bands, corroborating the picture that both bands have most of their weight on the same type of ion, specifically Cl^−^ in the case of NaCl (Fig. 3b).

## Discussion

Naively applying classical electrostatics, one would predict that the negatively charged electrons avoid the negatively charged Cl^−^ in favour of the positively charged Na^+^. Moreover, because Cl^−^ has a full shell electron configuration [Ne]3*s*^2^3*p*^6^, the extra tunnelling electron must be accommodated in the higher energy Cl 4*s* orbital, which also appears to impose an insurmountable energetic cost.

To understand why the Cl 4*s* states might, nevertheless, be so important, we make a few very simple considerations. For fully ionised atoms (in the sense of Cl^−^ and Na^+^ having exact full shell electronic configurations), the Madelung potential is 8.9 eV, leading to a strong upward shift of the Na 3*s* level, which for a neutral atom is just at −5.1 eV^30^. Even if the 3*s* orbital relaxes somewhat, it may then be pushed above the vacuum level by the Madelung potential.

Meanwhile, the 4*s* level of a free Cl^−^ ion is above the vacuum level. The issue is whether the Madelung potential can pull this level below the Na 3*s* level. To puzzle this out, we use a thought experiment to increase the nuclear charge of Cl by two. This turns Cl^−^ into K^+^, for which the 4*s* level is at −4.1 eV^30^, below the (raised) Na 3*s* level. The question is if the Madelung potential has a similar attractive effect on the Cl 4*s* level. We use Slater’s rules^31^ to approximate the 4*s* state of K^+^ as *ϕ*_4*s*_ ~ *r*^2.7^ exp(−2.2*r*/3.7), where 2.2 is the effective nuclear charge and *n* = 3.7 is the effective principal quantum number. We then calculate the attractive potential on the 4*s* state due to increasing the nuclear charge of Cl by *ΔZ* = 2. The magnitude of this potential is indeed comparable to the Madelung potential of 8.9 eV. In reality, the atoms are not fully ionised and so the Madelung potential is smaller. Simple estimates suggest, however, that this effect and several other smaller effects do not change the picture in an essential way (see Supplementary Note 3). The K 4*s* orbital is very extended and complicates the interpretation of these results. Nevertheless, these simple considerations make it plausible that the Madelung potential can reverse the order of the Cl^−^ 4*s* level and the Na^+^ 3*s* level, resulting in a conduction band of mainly Cl character in NaCl. This is confirmed by the STM measurements in Figs. 1 and 2 that show well-defined features centred on the Cl ions. This interpretation is robust because the STM measurements are performed in real space and do not depend on interpretations expressed in terms of orbitals.

We now discuss differences between bulk NaCl and NaCl on Au, using tight-binding calculations described in the Supplementary Note 2. For a NaCl film on Au, we find an appreciable charge transfer to the Au substrate, consistent with an observed substantial reduction of the work function. This raises the potential of the outer layers of the film substantially, but the effect on the potential difference between the Na and Cl sites in the outermost layer is very small. The assumed smaller lattice parameter of the NaCl film on Au increases the Madelung potential, while the finite thickness of the film reduces it. Adding up all effects, we find that the tendency to put the conduction band on the Cl atoms should be comparable (difference on the order of 0.1 eV, see Supplementary Table IV) for bulk NaCl and for NaCl on Au, implying that the effects observed here should apply also to bulk NaCl.

In nanoscience, a NaCl film is often used as a buffer between a substrate and a molecule to be studied^32,33^. Due to the large NaCl gap, the applied voltage is typically such that the electrons tunnel through the NaCl gap. The way the substrate couples to the molecule then depends on whether the corresponding electrons mainly tunnel through the Cl^−^ or Na^+^ ions. The results above should then be important for interpreting such experiments.

In contrast to the discussion above, in nanoscience it is often assumed that the electrons tunnel through a band gap via some state, not well specified, being different from both the valence and conduction states^34^. To address this issue, we could have included additional basis states, which would then have to be orthogonal to the valence and conduction states of the NaCl film. However, such states would necessarily be higher in energy than the conduction states already included. This implies that the additional basis states would not be very important for describing gap states, which are located below the conduction band. By contrast, the explicit use of just valence and conduction states as basis states should already give a good qualitative picture of what happens when tunnelling through the gap, and such calculations are relatively easy to interpret.

In view of these results for Na^+^ Cl^−^ it is interesting to discuss Mg^2+^O^2−^. In analogy to the discussion above, we do the thought experiment of increasing the nuclear charge of O^2−^ by three, obtaining Na^+^ with its 3*s* level at −5.1 eV. The attractive interaction of this additional nuclear charge with the 3*s* level is 20 eV, comparable to the Madelung potential 23.9 eV for the ions Mg^2+^ and O^2−^. This suggests that the conduction band of MgO could be located on oxygen, involving mainly O 3*s* levels. Indeed, this is what was found in band structure calculations by de Boer and de Groot^35^. It would then be very interesting to check experimentally the character of the conduction bands both for the II–VI and for other I–VII ionic compounds.

Beyond these simple salts, it would indeed be fruitful to further experiment with materials whose valence and conduction bands take on unusual combinations of cationic and anionic character, e.g., that of the valence band being cationic^36^, or that of the conduction band being anionic^37^. There may also be complementary mechanisms to probe that induce electronic structure rearrangements like that of the Madelung potential.

To summarise, using STM we have shown that the conduction band of NaCl has mainly Cl character, contrary to widespread belief. That the conduction band can be anionic in character is related to the large Madelung potential, which pushes the Na 3*s* level upwards and pulls the Cl 4*s* level downwards, which for a free Cl^−^ species is above the vacuum level. We provided a back of the envelope calculation to make it plausible that the Madelung potential could actually reverse the order of the Cl^−^ 4*s* and the Na^+^ 3*s* levels, leading to this result. This electronic structure may couple into other physical processes. Incidentally, it is the negative ion vacancies that are important to electrical conduction near the melting point of some alkali halides^38^.

## Methods

### Sample preparation and details of STM measurements

The experiments were carried out with a home-built low-temperature STM operated at *T* = 4.3 K in ultra-high vacuum (<10^−11^) mbar)^39^. The Au(111) single-crystal (>99.999%) sample was cleaned by repeated cycles of Ar^+^ ion sputtering at 10^−6^ mbar range argon pressure with 600 eV acceleration energy and subsequent annealing to 873 K. The sample heating and cooling rate was about 1 K/s. NaCl was evaporated thermally from a Knudsen cell held at 900 K, with the Au(111) surface held at 300 K, to obtain defect-free, (100)-terminated NaCl islands. An electrochemically etched gold wire^40^ (99.95% purity) was used as tip in the experiment. To ensure a metallic tip, the Au wire is further prepared by controlled tip indentations (1–3 nm, *V* = 50–100 mV) in Au(111) until atomic resolution is obtained at tunnelling current set point: *I*_*T*_  = 10 pA, +1 V. The text always specifies bias voltages of the metal substrate with respect to the grounded tip. Differential conductance (d*I/*d*V*) spectra were measured using a standard lock-in technique with a bias modulation of *V*_rms_ = 4 mV (Fig. 1b inset) and *V*_rms_ = 10 mV (Fig. 1b) at 629 Hz. Scanning tunnelling microscopy/spectroscopy data were analysed using self-written MATLAB code.

## Supplementary information

Supplementary Information

## Data Availability

The data generated in this study have been deposited in the figshare database under accession code 10.6084/m9.figshare.17159228^41^.
