# Supplementary material for: Anionic character of the conduction band of sodium chloride
Source: Nat Commun. 2022 Feb 21;13:981. doi: 10.1038/s41467-022-28392-8 (PMC8861091; doi:10.1038/s41467-022-28392-8)
Supplement: Supplementary file 1 — Supplementary Information [file 41467_2022_28392_MOESM1_ESM.pdf]

Supplementary Information
Anionic Character of the Conduction Band of Sodium Chloride

Christopher C. Leon,[1,*] Abhishek Grewal,[1,*] Klaus Kuhnke,[1,†] Klaus Kern,[1,2] and Olle Gunnarsson[1,‡]

[1] *Max-Planck-Institut für Festkörperforschung, Heisenbergstraße 1, 70569 Stuttgart, Germany*
[2] *Institut de Physique, École Polytechnique Fédérale de Lausanne, 1015 Lausanne, Switzerland*

SUPPLEMENTARY NOTE 1: PARAMETERS

Below we describe the parameters used to calculate the gap states in the NaCl film for the two cases when the conduction band is mainly on the Na atoms or mainly on the Cl atoms. We consider three NaCl(100) layers on five Au(111) layers, using periodic boundary conditions parallel to the surface.

Au

To describe Au, the hopping parameters of Harrison [1] were used. We also used the Harrison level energies $\varepsilon_{6s} = -6.98$ eV and $\varepsilon_{5d} = -17.78$ eV as a starting point. A $6p$ level at $\varepsilon_{6p} = -2.8$ eV was added. We then required that the top ($\bar{M}$-point) of the $5d$ band is placed at 1.7 eV below the Fermi energy [2]. This required a shift of the $5d$ level to $\varepsilon_{5d} = -14.33$ eV. These parameters are summarized in Supplementary Table I.

NaCl

To describe NaCl, we also used the hopping parameters of Harrison [1]. Two cases were considered. First we followed Harrison [1] and used the parameters $\varepsilon_{\mathrm{Na},3s} = -4.96$ eV, $\varepsilon_{\mathrm{Cl},3s} = -29.2$ eV and $\varepsilon_{\mathrm{Cl},3p} = -13.78$ eV. A Na $3p$ state was also added ($\varepsilon_{\mathrm{Na},3p} = -1$ eV). The effects of the Madelung potential were neglected. This led to a somewhat too large gap compared with the experimental result 8.5 eV [3]. The Na $3s$ level was therefore lowered somewhat to $\varepsilon_{\mathrm{Na},3s} = -5.55$ eV. These parameters are shown in Supplementary Table I as case 1.

Alternatively, we followed de Boer and de Groot [4] and assumed that the conduction band has Cl $4s$ character. For this purpose we replaced the Cl $3s$ level by a $4s$ level at $\varepsilon_{\mathrm{Cl},4s} = 1.35$ eV, leaving the Cl $3p$ level at $\varepsilon_{\mathrm{Cl},3p} = -13.78$ eV. We then took the Madelung potential into account and shifted the Na levels upwards ($\varepsilon_{\mathrm{Na},4s} = 4$ eV and $\varepsilon_{\mathrm{Na},3p} = 8$ eV). This also resulted in a band gap of 8.5 eV [3]. These parameters are shown in Supplementary Table I as case 2.

Element	Case	s	p	d
Au (6s, 6p, 5d)	1, 2	-6.98	-2.8	-14.33
Na (3s, 3p)	1	-5.55	-1.0	-
Cl (3s, 3p)	1	-29.2	-13.78	-
Na (3s, 3p)	2	4	8	-
Cl (4s, 3p)	2	1.35	-13.78	-

SUPPLEMENTARY TABLE I. Level positions used for NaCl on Au. Case 1 and 2 correspond to a conduction band of mainly Na $3s$ or Cl $4s$ character, respectively.

Combined system

For the hopping between the Au and NaCl slabs we introduced a cut off at a distance of $\sqrt{d^2_{\mathrm{Au-NaCl}} + a^2_{\mathrm{NaCl}}/2}$. The hopping integrals were then calculated according to the prescription of Harrison [1]. Then each Au atom in the outermost Au layer typically couples to about 5-7 atoms in the closest NaCl layer. We then shifted all the Au level energies so that the Fermi energy is at zero. Calculations using the GW method [5] find that for NaCl on Au, the top of the NaCl valence band is about 5 eV below the Fermi energy of Au [6]. We then shifted all NaCl levels correspondingly.

Calculations find that the lattice parameter of a three layer NaCl film on Au is reduced compared with bulk NaCl [7]. We therefore used the calculated reduced lattice parameter $a_{\mathrm{NaCl}} = 5.54$ Å [7]. We used the calculated separation $d_{\mathrm{Au-NaCl}} = 3.12$ Å between the Au surface and the NaCl film [7]. For Au we use the lattice parameter $a_{\mathrm{Au}} = 4.07$ Å [8].

SUPPLEMENTARY NOTE 2: COMPARISON OF A THIN NACL FILM ON AU AND BULK NACL

Bulk NaCl: In the main text, we used the Madelung potential for bulk NaCl and fully ionized atoms. This potential is given by

$$V = 1.7476 \frac{e^2}{4\pi\epsilon_0 d} = 8.92 \text{ V}, \quad (1)$$

where $d = a_{\mathrm{NaCl}}/2 = 2.82$ Å is the separation of the nearest neighbor Cl and Na atoms, ϵ_0 is the vacuum electric permeability and we assume full positive and negative elementary charges on Na and Cl, respectively. We now discuss the corrections to this, focusing on the differences between bulk NaCl and a thin NaCl film on Au.

Tight-binding calculations for bulk NaCl give the net charge 0.805 of the ions (see Supplementary Table II). This reduces the Madelung potential to 7.18 V. This is the reference for the following considerations. We now focus on the differences between bulk NaCl and a few layers of NaCl on an Au substrate.

Reduced lattice parameter: The lattice parameter of a NaCl film is assumed to be reduced to $a_{\mathrm{NaCl}} = 5.54$ Å [7]. This increases the Madelung potential by 0.129 V compared with the bulk. This is shown in Supplementary Table IV as a positive contribution under "Film lattice parameter smaller".

Na $3s$	Na $3p$	Net charge Na	Cl $4s$	Cl $3p$	Net charge Cl
0.069	0.126	0.805	0.004	5.801	-0.805

SUPPLEMENTARY TABLE II. Orbital weights and net charges on Na and Cl atoms for bulk NaCl.

Layer	1		2		3		4		Au
Layers	Na	Cl	Na	Cl	Na	Cl	Na	Cl	Subst.
2	.767	-.660	.834	-.834					-.107
3	.769	-.660	.800	-.805	.836	-.834			-.106
4	.769	-.660	.802	-.805	.802	-.805	.836	-.834	-.105
Bulk	.805	-.805							

SUPPLEMENTARY TABLE III. Net total charges on Na and Cl atoms in different layers of a film with 2-4 layers outside an Au substrate and net charge on the Au substrate per pair of Na and Cl atoms.

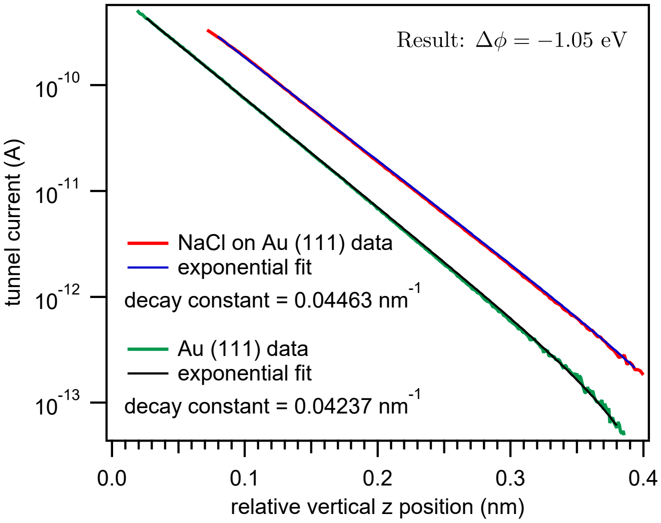

SUPPLEMENTARY FIGURE 1. Current vs distance measurements with the same tip on Au(111) and on a nearby 2 ML NaCl/Au(111). The measurements are performed at a bias voltage of -0.2 V. Forward and backward scans were averaged using the geometric average. The decay constants for k_{Au} and k_{NaCl} convert to a work function difference ($\Delta\phi$) as: $\Delta\phi = 0.01905(1/k^2_{\mathrm{NaCl}} - 1/k^2_{\mathrm{Au}})(\mathrm{nm}^2\mathrm{eV})$.

Film thickness: Due to the NaCl film being just a few layers, the Madelung potential of the film is reduced by about 0.27 V compared with the bulk. This is shown under "Finite film thickness" in Supplementary Table IV.

Charge densities and work function: We perform tight-binding calculations for a thin film of a few layers of NaCl on an Au substrate with three layers. Each layer has 324 atoms. The charges on the atoms in the layers are shown in Supplementary Table III. For 2-4 NaCl layers there is a net positive charge on the film of about 0.1 electrons per pair of Na and Cl atoms. Putting the corresponding charge at the center of the outermost Au layer leads to a large reduction of the work function by about 2.0 eV. Experimentally, the work function of Au is 5.33 eV[9], 5.3-5.6 eV [10] or 5.5 eV [11]. For NaCl on Au(111) the work function is reduced to 4.3 eV [12]. Using $I(z)$ spectroscopy, we measure a work function reduction of 1.05 eV (see Supplementary Figure 1) also suggesting a work function of $\approx$ 4.3 eV for NaCl on Au(111). This reduction is not quite as large as in the calculation. The deviation is not surprising, given that the tight-binding calculation only provides charges on atoms but no distortions of wave functions.

Increased charge on outermost layer: The hopping between a Cl atom and its neighboring Na atoms reduces the charge on Cl. This results in the occupancy of the $3p$ level of 5.801 for bulk NaCl, i.e., a reduction by 0.199 from the nominal value 6.0. In the outermost layer of a NaCl film, the number of Na neighbors is reduced from six to five. We then expect fewer Cl $3p$ holes than in the bulk, i.e., the number of holes to be reduced from 0.199 in the bulk to 0.199*(5/6)=0.166 in the film, about 0.033 smaller than in the bulk. This agrees fairly well with the calculated reduction of 0.029 for a two layer film. The Madelung potential for one single layer of fully ionized atoms is 8.39 V. The increase of the Madelung potential in the outermost layer due to the extra charge is then 0.029*8.39=0.243 V. For a three layer film we obtain the result $((0.836+0.834)/2-0.805)*8.39 = 0.252$ V. This is shown under "Increase of charge on the outermost layer" in Supplementary Table IV.

Reduced charge on inner layers: We first neglect the charge transfer to the Au substrate and temporarily put this charge on the Na atoms in the layer closest to Au. The charge transfer between Na and Cl is still slightly smaller on the inner layer than in bulk NaCl. This slightly reduces the Madelung potential on the atoms in the outermost layer, shown under "Change charge inner layers".

Charge transfer to Au: The charge transfer to Au from NaCl has a very large effect on the work function (1 eV or more). However, the work function refers to a uniform shift of the potential well outside the NaCl layers. Here we are interested in the difference in potential between Na and Cl atoms in the surface layer. This turns out to be very small for two layers and negligible for three layers (see "Charge transfer to Au"), although there is a large shift of the potential in the outermost layers.

Cl $3p-4s$ interaction: The increase in the $3p$ charge of the Cl atom at the surface of the film tends to raise the $4s$ level via the on-site Coulomb interaction. To address this, we use the similarity of a K^+ ion and a Cl^- ion in the Madelung potential, discussed in the main text. We use K^+ atomic data [13] to estimate the shift of the $4s$ level due to the extra $3p$ charge (0.029). This gives an upward shift of $0.029 \times 7.32 = 0.212$ eV, where 7.32 eV is the $3p-4s$ direct Coulomb integral, derived from atomic data. We assume a similar but opposite effect on the Na $3s$ level. This reduces the Na $3s$ and Cl $4s$ splitting, shown as "On-site Cl contribution in Supplementary Table IV. In the end, the increase of the charges in the outermost layer plays a rather small role, due to the approximate canceling of the on-site Coulomb interaction and the contribution to the Madelung potential.

We conclude that the lowering of the Cl $4s$ level relative

Effect	2 layers	3 layers
Film lattice parameter smaller	0.129	0.129
Finite film thickness	-0.276	-0.279
Increase of charge on the outermost layer	0.244	0.252
Change charge inner layers	-0.051	-0.001
Charge transfer to Au	0.019	0.000
On-site Cl contribution	-0.212	-0.212
Sum	-0.147	-0.111

SUPPLEMENTARY TABLE IV. Contributions to the difference of the Madelung potential (in V) for the outermost layer of a thin NaCl film outside an Au substrate compared with bulk NaCl. Positive values imply a larger Madelung potential for the NaCl film. We have also added the contribution from the on-site Coulomb integral due to the increased $3p$ charge on Cl atoms in the outermost layer. The negative total contribution implies that the Madelung potential is slightly larger in bulk NaCl.

to the Na $3s$ level is very similar in bulk NaCl and NaCl on an Au surface, and the effect may even be larger in bulk NaCl. The observation that the conduction band is mainly on the Cl atoms should then also apply to bulk NaCl.

SUPPLEMENTARY NOTE 3: I-VII AND II-VI COMPOUNDS

Here we discuss the possibilities that I-VII and II-VI compounds in general may have the conduction band on the anion. We calculate the Madelung potential for fully ionized atoms. The effect of the Madelung potential on an s-orbital outside a free, negatively charged, anion is not trivial to estimate, since for the free ion this orbital is not bound. Therefore we make the thought experiment of increasing the nuclear charge on the anion by $\Delta Z = 2$ for the I-VII compounds and by $\Delta Z = 3$ for the II-VI compounds, converting, e.g., Cl^- into K^+ or O^{--} into Na^+. This results in a bound s orbital outside a full shell. We can then easily estimate the expectation value of the potential from the extra nuclear charge for the bound s-orbital, e.g, the $4s$ orbital of K^+. This provides an estimate of the strength of the potential needed to bind the anion s-orbital. If the Madelung potential is comparable or larger, it seems plausible that it could have pulled the previously unbound s-orbital below the vacuum level. We calculate

$$\langle \phi(r) | \frac{1}{r} | \phi(r) \rangle, \tag{2}$$

where

$$\phi(r) \sim r^{n_{\rm eff}-1} e^{-(Z_{\rm eff}/n_{\rm eff})r}, \tag{3}$$

is the Slater orbital [14] of the alkali ion (e.g., K^+ for the case of NaCl) obtained by increasing the nuclear charge by ΔZ, and $Z_{\rm eff} = 2.2$ is the Slater effective nuclear charge for that orbital. The results are shown in Supplementary Table V. We then compare this integral times ΔZ with the Madelung potential for fully ionized atoms in Supplementary Table VI for a few ionic compounds. The Madelung potential is typically comparable to $\langle \phi(r) | \Delta Z/r | \phi(r) \rangle$, which suggests that the conduction band could be on the anion.

Anions	Alkali ion	$n_{\rm eff}$	$\langle \phi | 1/r | \phi \rangle$ [eV]
O^{--}, F^-	Na^+	3	6.7
S^{--}, Cl^-	K^+	3.7	4.4
Se^{--}, Br^-	Rb^+	4.0	3.7
Te^{--}, I^-	Cs^+	4.2	3.4

SUPPLEMENTARY TABLE V. Anions and and the alkali ion obtained by increasing the nuclear charge by ΔZ, the effective quantum number $n_{\rm eff}$ of the alkali ion as well as $\langle \phi | 1/r | \phi \rangle$, where ϕ is the alkali ion s-function.

We should, however, remember that the ions are not fully ionized, reducing the Madelung potential. The s-orbital outside the negatively charge anion is also quite extended, making the assignment of its charge to the anion somewhat questionable. On the other hand, the strong variation of the Madelung potential between the ions may be sufficient to also localize the anion s-orbital. The s-orbital on the cation is moved upwards, and it is sufficient that the s-orbital on the cation is well above the s-orbital outside the anion to obtain these results. The considerations above, however, can only be seen as suggestive and used to rationalize the rather surprising experimental results for NaCl and, possibly, for other ionic compounds. Decisive is that STM provides a real space measurement, which does not depend on a discussion in terms of orbitals and that STM for NaCl provides a well-defined image localized on the Cl atoms.

Compound	ΔZ	Madelung [eV]	$\langle \phi | \Delta Z/r | \phi \rangle$ [eV]
NaCl	2	8.9	8.8
LiI	2	8.3	6.8
RbF	2	8.9	13.4
MgO	3	23.9	20.1
BaO	3	18.2	20.1

SUPPLEMENTARY TABLE VI. Some ionic compounds and the ΔZ needed to convert the anion into an alkali ion. The table also shows the Madelung potential for fully ionized atoms and the attraction on the outermost anion s-orbital from the increase, ΔZ, in the nuclear charge. If these two quantities are comparable, it becomes plausible that the conduction band could be on the anion.

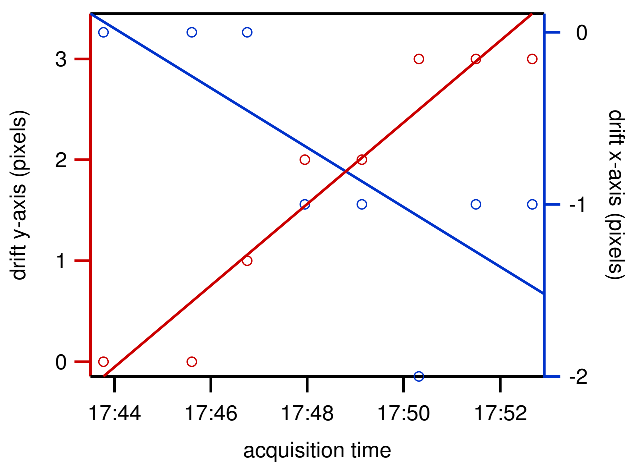

SUPPLEMENTARY FIGURE 2. Evaluation of drift for data in Fig. 2 in the main manuscript. 2.4×2.4 nm^2 sized topographs are recorded at resolution of 64×64 pixels. Total data acquisition time for the data set is 10 mins. From linear fit we get drift along x-axis: -2 pixels and along y-axis: 3 pixels. Total drift: 135 pm.

SUPPLEMENTARY REFERENCES

* C.C.L. and A.G. contributed equally to this work.
† k.kuhnke@fkf.mpg.de
‡ o.gunnarsson@fkf.mpg.de

[1] Walter A. Harrison. *Elementary Electronic Structure*. WORLD SCIENTIFIC, Singapore, 1999.

[2] P. M. Sheverdyaeva, R. Requist, P. Moras, S. K. Mahatha, M. Papagno, L. Ferrari, E. Tosatti, and C. Carbone. Energy-Momentum Mapping of d-Derived Au(111) States in a Thin Film. *Phys. Rev. B*, 93(3):035113, 2016.

[3] R. T. Poole, J. G. Jenkin, J. Liesegang, and R. C. G. Leckey. Electronic Band Structure of the Alkali Halides. I. Experimental Parameters. *Phys. Rev. B*, 11(12):5179–5189, 1975.

[4] P. K. de Boer and R. A. de Groot. The Origin of the Conduction Band in Table Salt. *Am. J. Phys.*, 67(5): 443–445, 1999.

[5] Lars Hedin. New Method for Calculating the One-Particle Green's Function with Application to the Electron-Gas Problem. *Phys. Rev.*, 139(3A):A796–A823, August 1965.

[6] Shiyong Wang, Neerav Kharche, Eduardo Costa Girão, Xinliang Feng, Klaus Müllen, Vincent Meunier, Roman Fasel, and Pascal Ruffieux. Quantum Dots in Graphene Nanoribbons. *Nano Lett.*, 17(7):4277–4283, 2017.

[7] Hsin-Yi Tiffany Chen and Gianfranco Pacchioni. Properties of two-dimensional insulators: A DFT study of Co adsorption on NaCl and MgO ultrathin films. *Phys. Chem. Chem. Phys.*, 16(39):21838–21845, 2014.

[8] Wheeler P. Davey. Precision Measurements of the Lattice Constants of Twelve Common Metals. *Phys. Rev.*, 25(6): 753–761, 1925.

[9] J. M. White. Surface Science Investigations of Thin Metal Films on Metal and Metal Oxide Supports. *MRS Online Proceedings Library*, 83(1):133–139, 1986.

[10] Jianwei Sun, Adrienn Ruzsinszky, and John P. Perdew. Strongly Constrained and Appropriately Normed Semilocal Density Functional. *Phys. Rev. Lett.*, 115(3):036402, 2015.

[11] B. Bröker, R.-P. Blum, J. Frisch, A. Vollmer, O. T. Hofmann, R. Rieger, K. Müllen, J. P. Rabe, E. Zojer, and N. Koch. Gold Work Function Reduction by 2.2eV with an Air-Stable Molecular Donor Layer. *Appl. Phys. Lett.*, 93(24):243303, 2008.

[12] Zhe Li, Hsin-Yi Tiffany Chen, Koen Schouteden, Ewald Janssens, Chris Van Haesendonck, Peter Lievens, and Gianfranco Pacchioni. Spontaneous Doping of Two-Dimensional NaCl Films with Cr Atoms: Aggregation and Electronic Structure. *Nanoscale*, 7(6):2366–2373, 2015.

[13] Charlotte Emma Moore. *Atomic energy levels*. National Bureau of Standards, 1971.

[14] J. C. Slater. Atomic Shielding Constants. *Phys. Rev.*, 36 (1):57–64, 1930.
